# Nigrodiquinone A, a Hydroanthraquinone Dimer Containing a Rare C-9–C-7′ Linkage from a Zoanthid-Derived *Nigrospora* sp. Fungus

**DOI:** 10.3390/md14030051

**Published:** 2016-03-09

**Authors:** Wei-Feng Xu, Xue-Mei Hou, Kai-Lin Yang, Fei Cao, Rui-Yun Yang, Chang-Yun Wang, Chang-Lun Shao

**Affiliations:** 1 xuweifeng_u@163.com; 2 houxuemei_1990@163.comyangkailin0228@163.comchangyun@ouc.edu.cn; 3 caofei542927001@163.com; 4

**Keywords:** zoanthid *Palythoa haddoni*, *Nigrospora* sp., hydroanthraquinone dimer, antibacterial, antiviral

## Abstract

One new hydroanthraquinone dimer with a rare C-9–C-7′ linkage, nigrodiquinone A (**1**), and four known anthraquinone monomers **2**–**5**, were isolated from a fungus *Nigrospora* sp. obtained from the zoanthid *Palythoa haddoni* collected in the South China Sea. The structure of **1** was established through extensive NMR spectroscopy, and the absolute configuration was elucidated by comparing computed electronic circular dichroism (ECD) and optical rotations (OR) with experimental results. All the compounds were evaluated for antiviral activity, and **1** was also evaluated for antibacterial activity. Compound **4** displayed mild antiviral activity against coxsackie virus (Cox-B3) with the IC_50_ value of 93.7 μM, and **5** showed an IC_50_ value of 74.0 μM against respiratory syncytial virus (RSV).

## 1. Introduction

Anthraquinone dimers are widespread and structurally-diverse family which have been isolated from marine-derived organisms including fungi, actinomycetes, tunicates and echinoderms [1,2,3,4,5,6]. They feature intriguing variety of linkages between the component anthraquinones. The structural features of these compounds may display different biological profiles. Bisanthraquinones with C-10–C-1′ and C-5a–C-2′ linkages obtained from *Streptomyces* sp., displayed potent methicillin resistant *Staphylococcus aureus* (MRSA) inhibiting activity [7]. Albopunctatone possessing a C-10–C-2′ connection isolated from the ascidian *Didemnum albopunctatum* showed equipotent activity towards chloroquines-ensitive and chloroquine-resistant strains of *Plasmodium falciparum* [8]. Another two dimmers, tetrahydroaltersolanol C and alterporriol Q featuring C-4–C-4′ and C-8–C-2′ linkages, isolated from a fungus *Alternaria* sp. exhibited obvious activity against the porcine reproductive and respiratory syndrome (PRRS) virus [9].

**Figure 1:**
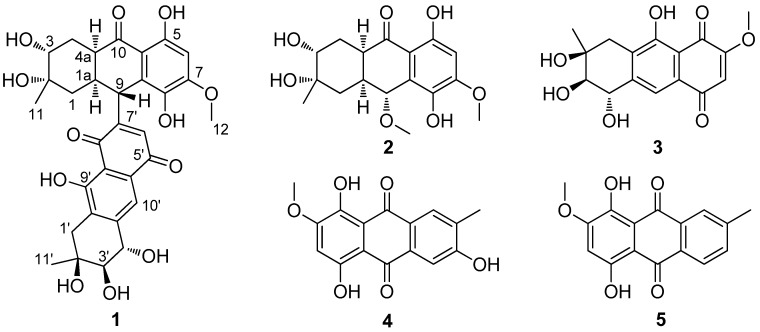
Chemical structures of **1**–**5**.

In our investigation of bioactive anthraquinone derivatives from marine-derived fungi, a series of anthraquinone monomers and anthraquinone dimers have been previously isolated from *Alternaria* sp. (ZJ-2008003) [9] and *Nigrospora* sp. (ZJ-2010006) [10]. Further chemical investigation of the fermentation broth of the *Nigrospora* sp. resulted in the isolation of a new hydroanthraquinone dimer, nigrodiquinone A (**1**), with a rare C-9–C-7′ linkage, together with four known hydroanthraquinone monomers, 4a-*epi*-9α-methoxydihydrodeoxybostrycin (**2**), 10-deoxybostrycin (**3**), 3,5,8-trihydroxy-7-methoxy-2-methylanthracene-9,10-dione (**4**), and austrocortirubin (**5**) (Figure 1). Herein, we report the isolation and structure elucidation of nigrodiquinone A (**1**), and antibacterial and antiviral activities of these five compounds.

## 2. Results and Discussion

Nigrodiquinone A (**1**) was isolated as a yellow amorphous powder. Its molecular formula C_31_H_32_O_12_ (sixteen degrees of unsaturation) was determined on the basis of HRESIMS data and combined with ^1^H and ^13^C NMR spectroscopic data. The deshielded area of ^1^H NMR spectrum (Table 1) revealed the presences of two hydrogen-bonded hydroxyl groups at δ_H_ 12.90 and 12.54 and three aromatic protons at δ_H_ 7.76, 6.51, and 6.13. Besides, one methoxy group (δ_H_ 3.93) and three oxymethine protons (δ_H_ 4.74, 3.54, and 3.31) were also observed by the ^1^H NMR spectrum analysis. Three carbonyl (δ_C_ 203.5, 189.9, and 183.7), six oxygen quaternary (δ_C_ 159.6, 159.1, 155.7, 136.0, 70.9, and 70.6), and three oxymethine (δ_C_ 77.4, 71.5, and 71.0) carbons were presented in **1** on the basis of careful analysis on the ^13^C NMR (Table 1) and DEPT spectra.

The ^1^H and ^13^C NMR data were suggested that **1** was a dimeric compound of 4a-*epi*-9α-methoxydihydrodeoxybostrycin (**2**) and 10-deoxybostrycin (**3**) [10]. The chemical shifts of C-9 and C-7′ were shifted to upfield (δ_C_ 36.8 and 129.7 in **1** *vs.* 72.8 in **2** and 160.9 in **3**) and the absence of two methoxy groups (δ_H_ 3.38 in **2** and δ_H_ 3.86 in **3**) indicated that this two monomers could be linked between C-9 and C-7′. Furthermore, the HMBC correlations from H-9 to C-6′, C-8′, and H-6′ to C-9 further confirmed that two units were joined via a C-9–C-7′ linkage. To the best of our knowledge, this is the first time to report the anthraquione dimer with a C-9–C-7′ linkage.

**Figure 2:**
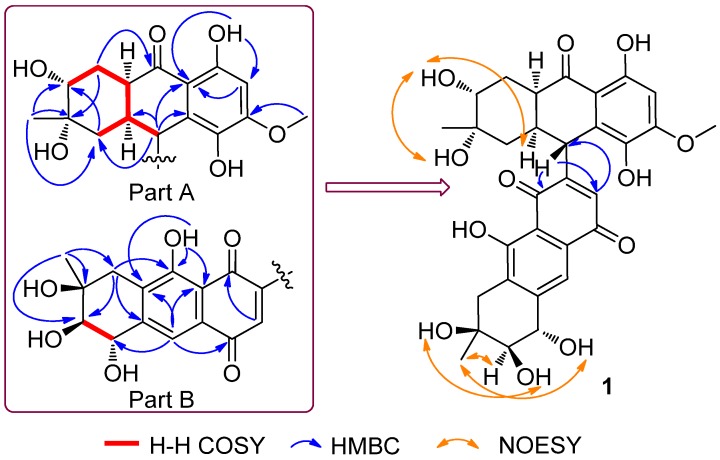
Key correlations for compound **1**.

In the selective 1D NOE experiments, the irradiation of H-9 resulted in no obvious enhancement of H-1a suggested that H-9 and H-1a might be *trans* oriented. The irradiation of H-3′ resulted in enhancement of H-11′ and no obvious enhancement of H-4′, and the key NOESY data (Figure 2) indicated that the relative configurations of all asymmetric carbons in **1** should be identical to those of **2** and **3** [10]. Thus, the relative configuration of **1** was determined as (1aR*,2S*,3R*,4aR*,9R*,2′S*,3′R*, and 4′S*).

**Figure 3:**
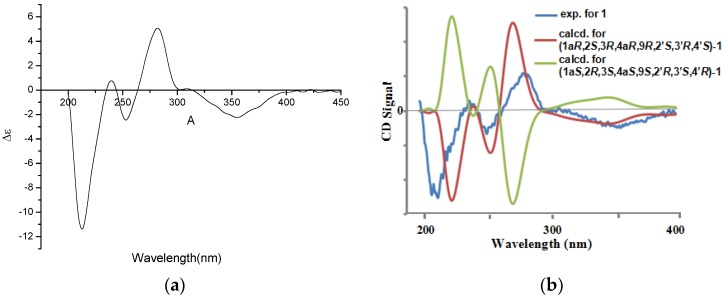
(**a**) Experimental CD spectra for **1** and (**b**) calculated electronic circular dichroism (ECD) spectra of **1**.

To compare computed electronic circular dichroism (ECD) and optical rotations (OR) with experimental results is a valid method to assign absolute configurations of natural products [11,12,13,14,15,16]. Thus, the absolute configuration of (**1**) was investigated by quantum chemical TDDFT calculations of their ECD and optical rotations (OR) spectra. ECD computations for four possible absolute configurations ((1a*R*,2*S*,3*R*,4a*R*,9*R*,2′*S*,3′*R*,4′*S*)-**1**, (1a*S*,2*R*,3*S*,4a*S*,9*S*,2′*R*,3′*S*,4′*R*)-**1**, (1a*R*,2*S*,3*R*,4a*R*,9*R*,2′*R*,3′*S*,4′*R*)-**1**, and (1a*S*,2*R*,3*S*,4a*S*,9*S*,2′*S*,3′*R*,4′*S*)-**1**) of **1** were carried out at the B3LYP/6-311G+(2d, p) level in the gas phase. Only the predicted ECD for (1a*R*,2*S*,3*R*,4a*R*,9*R*,2′*S*,3′*R*,4′*S*)-**1** look similar to the experimental result of **1** (Figure 3, and supporting information). The computed ORs in the gas phase were −39.1 for (1a*R*,2*S*,3*R*,4a*R*,9*R*,2′*S*,3′*R*,4′*S*)-**1**, +38.7 for (1a*S*,2*R*,3*S*,4a*S*,9*S*,2′*R*,3′*S*,4′*R*)-**1**, respectively, and the experimental optical rotation value was −36.0. Based on both of ECD and OR, the absolute configuration of **1** was assigned as 1a*R*,2*S*,3*R*,4a*R*,9*R*,2′*S*,3′*R*,4′*S*.

With regard to the connecting positions of monomers in the dimmers, the C-1–C-1′ [6], C-1–C-5′ [6], C-2–C-2′ [17], C-4–C-4′ [8], C-4–C-6′ [8], C-5–C-5′ [18], C-5–C-7′ [6], C-7–C-5′ [19], and C-8–C-8′ [8] connections have been reported to present in anterporriols family. Besides, the C-9–C-9′ [20], C-10–C–2′ [8], and C-10–C-1′/C-4a–C-2′ [7] linkages also presented in the anthraquinone dimmers. Nigrodiquinone A (**1**) is the first report to possess the rare C-9–C-7′ linkage.

Compounds **1**−**5** were evaluated for antiviral activity against Cox-B3 and RSV, with ribavirin as a positive control (Table 2). Compound **4** displayed mild antiviral activity against Cox-B3 with the IC_50_ value of 93.7 μM, and **5** showed an IC_50_ value of 74.0 μM against RSV. Compound **1** was evaluated for antibacterial activity against nine bacterial strains, Gram-positive *B. subtilis*, *B. cereus*, *M. luteus*, *M. tetragenus*, *S. albus*, *S. aureus*, Gram-negative *E. coli*, *V. anguillarum*, and *V. parahemolyticus*, however **1** was inactive at the concentration of 50 μM.

## 3. Materials and Methods

### 3.1. General Experimental Procedures

Optical rotations were measured on a JASCO P-1020 digital polarimeter. CD spectra were recorded on a MOS-450 spectrometer. IR spectra were recorded on a Bruker EQUINOX 55 spectrometer using KBr pellets. ^1^H and ^13^C spectra were recorded on a JEOL Eclips-600 spectrometer at 600 MHz for ^1^H and 150 MHz for ^13^C in DMSO-*d*_6_. Chemical shifts δ are reported in ppm, using TMS as internal standard and coupling constants (*J*) are in Hz. HRESIMS were measured on a Thermo MAT95XP High Resolution mass spectrometer. Silica gel (Qing Dao Hai Yang Chemical Group Co., Qingdao, China; 200–300 mesh), octadecylsilyl silica gel (Unicorn; 45–60 μm) and Sephadex LH-20 (Amersham Biosciences, Uppsala, Sweden) were used for column chromatography (CC). Precoated silica gel plates (G60, F-254, Yan Tai Zi Fu Chemical Group Co., Qingdao, China) were used for thin layer chromatography (TLC). Semi-preparative HPLC was performed on a Waters 1525 system using a semi-preparative C18 (Kromasil 7 μm, 10 × 250 mm) column coupled with a Waters 2996 photodiode array detector, at a flow rate of 2.0 mL/min.

### 3.2. Fungal Material

The fungal strain *Nigrospora* sp. (ZJ-2010006, Genbank NO. HM565952) [10] was isolated from a piece of fresh tissue from the inner part of the zoanthid *Palythoa haddoni* (GX-WZ-20100026) [21], collected from the Weizhou coral reefs in the South China Sea in April 2010. The strain was deposited at the Key Laboratory of Marine Drugs, the Ministry of Education of China, School of Medicine and Pharmacy, Ocean University of China, Qingdao, PR China. The fungal strain was cultivated in potato glucose liquid medium (composition of medium: 200 g/L cooked and sliced potatoes, 20 g/L glucose in artificial seawaters, in 1L Erlenmeyer flasks each containing 400 mL of culture broth) at 27 °C without shaking for 4 weeks.

### 3.3. Extraction and Isolation

The culture (80 L) was filtered to separate the culture broth from the mycelia. The culture broth was extracted with an equal volume of EtOAc and the fungal mycelia were extracted with CHCl_3_-MeOH (1:1, *v/v*) for three times, respectively. The organic extracts were evaporated under vacuum, and then the crude extract was suspended in H_2_O and partitioned with EtOAc. All the EtOAc layers were combined and evaporated to dryness under reduced pressure to give an EtOAc extract (98.4 g), which was subjected to silica gel column chromatography using gradient mixtures of petroleum ether (PE)/EtOAc (EA) (100% PE, 60:40, 40:60, 20:80, and 100% EA) to yield five fractions (Fr. 1–Fr. 5). Fr. 2 was subjected to silica gel column chromatography (CHCl_3_/MeOH) to obtain Fr. 2-1 (20:1, *v/v*) and Fr. 2-2 (10:1, *v/v*). Fr. 2-1 was repeated subjected to silica gel column chromatography to get **5** (7.2 mg). Fr. 2-2 was subjected to Sephadex LH-20 chromatography with mixtures of CHCl_3_-MeOH (1:1, *v/v*), purified by ODS column chromatography and preparative by HPLC on a C18 column (Kromasil, 7 μm, 10 × 250 mm, 2 mL/min) to obtain **2** (MeOH-H_2_O (75:25, *v/v*), 5.3 mg), **3** (MeOH-H_2_O (55:45, *v/v*), 12.5 mg) and **4** (MeOH-H_2_O (55:45, *v/v*), 50.1 mg). Fr. 3 was further purified by Sephadex LH-20 chromatography with mixtures of CHCl_3_-MeOH (1:1, *v/v*), and then semi-preparative HPLC, eluting with MeOH-H_2_O (50:50, *v*/*v*) to yield **1** (1.5 mg).

Nigrodiquinone A (**1**): yellow amorphous powder; [α]${}_{D}^{24}$ −36.0 (*c* 0.025, acetone); CD (0.84 mmol, MeOH) λ_max_ (*Δ*ε) 212 (−3.97), 239 (0.16), 254 (−1.00), 283 (1.79), 354 (−0.78) nm. IR (KBr) ν_max_ 3407, 2928, 1631, 1294 cm^–1^; mp 226 °C; ^1^H NMR and ^13^C NMR see Table 1; ESIMS *m/z* 597.1 [M + H]^+^; HRESIMS *m*/*z* 597.1975 (calcd for C_31_H_33_O_12_, 597.1972 [M + H]^+^).

The structures of **2**–**5** were assigned by spectroscopic methods and comparison of the ^1^H- and ^13^C NMR data with those reported in literature [10].

### 3.4. Biological Assays

Antiviral activity was evaluated by the cytopathic effect (CPE) inhibition assay according to established procedures, with ribavirin as a positive control [22]. Two viruses, coxsackie virus (Cox-B3) and respiratory syncytial virus (RSV) were used. Antibacterial activities were evaluated by the method as described previously [10]. Nine bacterial strains, Gram-positive *Bacillus cereus* (ACCC 11077), *Bacillus subtilis* (ATCC 6633), *Micrococcus luteus* (ATCC 49732), *Micrococcus tetragenus* (ATCC 13623), *Staphylococcus albus* (ATCC 8799), *Staphylococcus aureus* (ATCC 27154), Gram-negative *Escherichia coli* (ATCC 25922), *Vibrio anguillarum* (ATCC 19019), and *Vibrio parahemolyticus* (ATCC 17802) were used, and ciprofloxacin was used as a positive control.

### 3.5. Computational Section

Conformational searches were performed using MMFF94S force field for (1a*R*,2*S*,3*R*,4a*R*,9*R*,2′*S*,3′*R*,4′*S*)-**1**, (1a*S*,2*R*,3*S*,4a*S*,9*S*,2′*R*,3′*S*,4′*R*)-**1**, (1a*R*,2*S*,3*R*,4a*R*,9*R*,2′*R*,3′*S*,4′*R*)-**1**, and (1a*S*,2*R*,3*S*,4a*S*,9*S*,2′*S*,3′*R*,4′*S*)-**1**. All geometries (15 lowest energy conformers for (1a*R*,2*S*,3*R*,4a*R*,9*R*,2′*S*,3′*R*,4′*S*)-**1**, 15 for (1a*S*,2*R*,3*S*,4a*S*,9*S*,2′*R*,3′*S*,4′*R*)-**1**, 15 lowest energy conformers for (1a*R*,2*S*,3*R*,4a*R*,9*R*,2′*R*,3′*S*,4′*R*)-**1**, and 15 for (1a*S*,2*R*,3*S*,4a*S*,9*S*,2′*S*,3′*R*,4′*S*)-**1**, respectively) with relative energy from 0–10 kcal/mol used in optimizations at the B3LYP/6-311+G(d) level using Gaussian 09 package [23]. The B3LYP/6-311+G(d)-optimized conformers (3 lowest energy conformers for (1a*R*,2*S*,3*R*,4a*R*,9*R*,2′*S*,3′*R*,4′*S*)-**1**, 4 for (1a*S*,2*R*,3*S*,4a*S*,9*S*,2′*R*,3′*S*,4′*R*)-**1**, 3 lowest energy conformers for (1a*R*,2*S*,3*R*,4a*R*,9*R*,2′*R*,3′*S*,4′*R*)-**1** and 4 for (1a*S*,2*R*,3*S*,4a*S*,9*S*,2′*S*,3′*R*,4′*S*)-**1**; see Supporting Information for details) with relative energy from 0 to 4.6 kcal/mol were then re-optimized at the B3LYP/6-311+G(d) level. ECD and OR computations for all conformers were carried out at the B3LYP/6-311G+(2d, p) level in the gas phase. Boltzmann statistics were performed for ECD simulations with standard deviation of σ 0.16 eV.

## 4. Conclusions

As part of our ongoing investigation on discovering for biological secondary metabolites from zoanthid-derived fungi in the South China Sea, a new hydroanthraquinone dimer with a rare C-9–C-7′ linkage, nigrodiquinone A (**1**), was isolated from the culture of *Nigrospora* sp. The absolute configuration of nigrodiquinone A was determined by quantum chemical TDDFT calculations of theirs ECD and OR spectra. Compound **4** displayed mild antiviral activity against Cox-B3 with the IC_50_ value of 93.7 μM, and **5** showed an IC_50_ value of 74.0 μM against RSV.

## Figures and Tables

**Table 1:** NMR spectroscopic data of **1**.^a^

Position	δ_H_ (*J* in Hz)	δ_C_, Mult.	H–H COSY	HMBC
1	1.48, t, (13.5) 1.88, ddd, (13.5, 3.6, 2.3)	41.5, CH_2_	H-1a	C-3
1a	2.86, m	34.5, CH	H-1, 4a, 9	
2		70.6, C		
3	3.31, m	71.0, CH	H-4	
4	1.65, ddd, (12.6, 12.0, 4.8) 2.52, ddd, (12.7, 4.5, 2.7)	36.8, CH_2_	H-3, 4a	C-2, 10
4a	3.07, m	41.5, CH	H-1a, 4	
5		159.1, C		
6	6.51, s	98.7, CH		C-10a
7		155.7, C		
8		136.0, C		
9	4.68, br s	36.8, CH		C-1, 1a, 9a, 10a, 6′, 8′
9a		124.9, C		
10		203.5, C		
10a		109.6, C		
11	1.17, s	26.8, CH_3_		C-1, 2, 3
12	3.93, s	55.8, CH_3_		C-7
1′	2.68, d (19.0), 3.07, d (19.0)	36.8, CH_2_		C-3′, 4a′, 9′
1a′		130.2, C		
2′		70.9, C		
3′	3.54, d (8.7)	77.4, CH	H-4′	
4′	4.74, br d (8.7)	71.5, CH	H-3′	
4a′		148.1, C		
5′		183.7, C		
6′	6.13, d (1.1)	135.3, CH		C-8′, 9
7′		129.7, C		
8′		189.9, C		
9′		159.6, C		
9a′		113.0, C		
10′	7.76, br s	117.3, CH		C-1a′, 4′, 5′, 9a′
10a′		100.0, C		
11′	1.43, s	26.3, CH_3_		C-1′, 2′, 3′
2-OH	3.17, br s			
3-OH	3.71, d (4.9)			
5-OH	12.90, s			C-6, 10a
8-OH	7.61, s			
2′-OH	3.67, br s			
3′-OH	4.27, br s			
4′-OH	4.87, br s			
9′-OH	12.54, s			C-1a′, 9′, 9a′

^a^ Measured at 600 MHz for ^1^H NMR and 150 MHz for ^13^C NMR in acetone-*d*_6_.

**Table 2:** Antiviral activities of compounds **1**−**5**. ^a^

**Virus**	**IC_50_ (μM)**					
	1	2	3	4	5	Ribavirin ^b^
RSV	-	-	-	-	74.0	78.0
Cox-B3	-	-	-	93.7	-	39.0

^a^ Data are expressed in IC_50_ values (*μ*M). ^b^ Ribavirin was used as a positive control. “-” means no antiviral activities.
